# Multiple-trait model through Bayesian inference applied to *Jatropha curcas* breeding for bioenergy

**DOI:** 10.1371/journal.pone.0247775

**Published:** 2021-03-04

**Authors:** Marco Antônio Peixoto, Jeniffer Santana Pinto Coelho Evangelista, Igor Ferreira Coelho, Rodrigo Silva Alves, Bruno Gâlveas Laviola, Fabyano Fonseca e Silva, Marcos Deon Vilela de Resende, Leonardo Lopes Bhering

**Affiliations:** 1 Universidade Federal de Viçosa, Viçosa, Minas Gerais, Brazil; 2 Instituto Nacional de Ciência e Tecnologia do Café, Universidade Federal de Viçosa, Viçosa, Minas Gerais, Brazil; 3 Embrapa Agroenergia, Brasília, Federal District, Brazil; 4 Embrapa Café, Universidade Federal de Viçosa, Viçosa, Minas Gerais, Brazil; Graduate University of Advanced Technology, Kerman Iran, ISLAMIC REPUBLIC OF IRAN

## Abstract

Multiple-trait model tends to be the best alternative for the analysis of repeated measures, since they consider the genetic and residual correlations between measures and improve the selective accuracy. Thus, the objective of this study was to propose a multiple-trait Bayesian model for repeated measures analysis in *Jatropha curcas* breeding for bioenergy. To this end, the grain yield trait of 730 individuals of 73 half-sib families was evaluated over six harvests. The Markov Chain Monte Carlo algorithm was used to estimate genetic parameters and genetic values. Genetic correlation between pairs of measures were estimated and four selective intensities (27.4%, 20.5%, 13.7%, and 6.9%) were used to compute the selection gains. The full model was selected based on deviance information criterion. Genetic correlations of low (*ρ*_*g*_ ≤ 0.33), moderate (0.34 ≤ *ρ*_*g*_ ≤ 0.66), and high magnitude (*ρ*_*g*_ ≥ 0.67) were observed between pairs of harvests. Bayesian analyses provide robust inference of genetic parameters and genetic values, with high selective accuracies. In summary, the multiple-trait Bayesian model allowed the reliable selection of superior *Jatropha curcas* progenies. Therefore, we recommend this model to genetic evaluation of *Jatropha curcas* genotypes, and its generalization, in other perennials.

## Introduction

The increasing energy demand and environmental awareness about pollution caused by fossil fuels have raised the interest of private companies, governments, and agencies of many countries in the development and production of alternative and sustainable energy sources. The use of bioenergy has been pointed out in the last decades as one of many alternatives to reduce the need for fossil fuels. Bioenergy should be interpreted as a renewable source of biomass, *i*.*e*. sugar/lignocellulosic tissues and vegetable oils [1]. Many crops have been identified as potential feedstock to obtain biofuels, mainly corn, sugar cane, soybean, castor bean oil, forest biomass, livestock manure, oil palm, peanut, canola and physic nut (*Jatropha curcas* L.) [2]. Then, biofuel may be used in engines either directly as fuel or as a blend mixed with fossil fuels [3, 4].

The energetic worldwide matrix emerges for the consumer of non-renewable fuels, when compared with the Brazilian scenario [5]. Brazil has applied extensive efforts in research and production of such fuels. In this country, *Jatropha curcas* has been indicated as an important alternative to the increasing demand for oil production, an important source for biofuel and bio kerosene [6, 7]. This species grabs attention due to its potential for oil production, high adaptability in a range of environments, tolerance to drought and longevity. It is also an alternative for diversifying the biofuel sector [5, 8, 9].

As a perennial, *Jatropha curcas* provides several records over time in the same experiment, thus characterizing repeated measures data. This kind of data demands efficient statistical methodologies to infer on the accuracy of the estimated genetic values and gains with selection. Studies carried out under such framework denote several alternatives to point out for correlation structure between measurements (including compound symmetry models, random regression models, and multiple-trait models—MTM).

The MTM has been indicated as the most effective procedure for repeated measure analysis in perennials breeding. Its efficiency is due to the use of the genetic and residual correlations between the repeated measures. Besides that, MTM allows the estimation of variance components and genetic values for each measure [10, 11].

In general, Bayesian inference has outperformed traditional frequentist analyses [12–15] by: (i) providing additional results to the frequentist approach, such as creditability and highest posterior density intervals (HPD); (ii) estimating the genetic parameters and the genetic values with increased precision, once it is conducted by Gibbs sampler, which is a Markov Chain Monte Carlo (MCMC) sampling algorithm; and, (iii) being a flexible methodology that allows to estimate accurately variance components and genetic values, even from reduced samples.

The MTM has been already applied to *Jatropha curcas* breeding [10, 16], but no study has addressed the Bayesian MTM for repeated measures analysis. In this context, the objective of this study was to propose the Bayesian MTM for repeated measures analysis in *Jatropha curcas* breeding for bioenergy.

## Material and methods

### Experimental design and genetic material

The experiment was implemented in November 2008, in the experimental field of Embrapa Cerrados, Planaltina − DF, Brazil (15°35′30” S and 47°42′30” W; 1007 m asl), as a randomized block design, with two replicates and five plants per plot. The plants were arranged in rows, spaced 4 m between rows and 2 m between plants. All management practices were based on [17, 18]. The experiment consisted of a performance evaluation of 730 individuals of 73 half-sib families. Each family was derivate from one tree (female parental), that received pollen from the whole population, given that *Jatropha curcas* is a allogamous plant [5]. The plants were evaluated for grain yield (kg plant^−1^) trait over six harvests, from 2010 to 2015 crop years.

### Statistical analyses

The Bayesian MTM, considering the experimental design, was given by:

y=Xβ+Zg+Wp+e,

where ***y*** is the vector of phenotypic data, with the conditional distribution assumed as: **y** | **β, g, p**, **G**, **T**, **R** ~ **N(Xβ + Zg + Wp, R ⊗ I)**, where **G** is the family (co)variance matrix, **T** is the plot (co)variance matrix, **R** is the residual (co)variance matrix, and **I** is an identity matrix; ***β*** is the vector of block effects (systematic effect) added to the overall mean; ***g*** is the vector of family effects, assumed as **g|G ~ N(0, G ⊗ I)**; ***p*** is the vector of plot effects, assumed as **p|T ~ N(0, T ⊗ *I*)**; **e** is the vector of residual effects, assumed as **e|R ~ N(0, R ⊗ *I*)**. The terms X, Z, and W refers to the incidence matrix for the block, family, and plot effects, respectively.

We assumed that **G, T** and **R** follow an inverted Wishart distribution WI (**v**, **V**), with hyperparameters **v** and **V** [19]. The hyperparameters for all prior distributions were selected to provide non-informative or flat prior distribution. For the systematic effect (***β*)**, a uniform prior distribution was attributed. Furthermore, the parameters ***β***, ***g***, ***p***, **G**, **T**, and **R** were estimated, following the joint posteriori distribution: P(***β***, ***g***, ***p***, **G**, **T**, **R**|y) α P(y | ***β***, ***g***, ***p***, **G**, **T**, **R)** × P(***β***, ***g***, ***p***, **G**, **T**, **R)**.

Variance components and genetic parameters were estimated by the Gibbs sampling algorithm. The family variances (${\hat{\sigma }}_{g}^{2}$) were obtained from the diagonal of the estimated matrix **G**, the plot variances (${\hat{\sigma }}_{p}^{2}$) were obtained from the diagonal of the estimated matrix **T**, and the residual variances (${\hat{\sigma }}_{res}^{2}$) were obtained from the diagonal of the estimated matrix **R**. We consider a total of 10,000,000 interactions, with burn-in of the 1,000,000 and sampling interval period (thin) of the 50. This led to a sample of the 180,000 iterations.

All effects from the model were tested against the full model by removing one effect (genetic and plot) at a time and calculating the deviance information criterion (DIC). As [20], the DIC was described as: DIC = D(θ) + 2pD, where D(θ) is a point estimate of the deviance obtained by replacing the parameters with their posterior mean estimates in the likelihood function and pD is the effective number of parameters in the model. The convergence of the parameters was checked by the Geweke criterion [21]. Posterior means and high posterior probability (HPD) for estimated parameters were obtained from MCMC samples.

The estimates of additive genetic variance between families (${\hat{\sigma }}_{a}^{2}$), individual phenotypic variance (${\hat{\sigma }}_{phen}^{2}$), heritability of family means ($h_{g}^{2}$), coefficient of determination of plot effect ($c_{p}^{2}$), coefficient of determination of residual effects ($c_{res}^{2}$) and Bayesian selective accuracy ($r_{\hat{g}g}$) were obtained as follows:

$${\hat{\sigma }}_{a}^{2}={4\hat{\sigma }}_{g}^{2},$$

$${\hat{\sigma }}_{phen}^{2}={\hat{\sigma }}_{g}^{2}{+\hat{\sigma }}_{p}^{2}+{\hat{\sigma }}_{res}^{2},$$

$$h_{g}^{2}=\frac{{\hat{\sigma }}_{g}^{2}}{{\hat{\sigma }}_{g}^{2}+\frac{1}{r}{\hat{\sigma }}_{p}^{2}+\frac{1}{nr}{\hat{\sigma }}_{res}^{2}},$$

$$c_{p}^{2}=\frac{{\hat{\sigma }}_{p}^{2}}{{\hat{\sigma }}_{phen}^{2}},$$

$$c_{res}^{2}=\frac{{\hat{\sigma }}_{res}^{2}}{{\hat{\sigma }}_{phen}^{2}},$$

and

$$r_{\hat{g}g}=1-\frac{S(g)}{GV},$$

where *n* is the number of individuals in the plot (five individuals), *r* is the number of repetitions (two repetitions), and *S(g)* is the standard deviations of the estimated genetic values (GV).

Genetic correlations (*ρ*_*g*_) and phenotypic correlations (*ρ*_*p*_) between each pair of measures were calculated as follows:

$$\rho_{g}=\frac{{\hat{\sigma }}_{g(ij)}}{\sqrt{{\hat{\sigma }}_{g(i)}^{2}{\hat{\sigma }}_{g(j)}^{2}}},$$

and

$$\rho_{p}=\frac{{\hat{\sigma }}_{g(ij)}{\hat{\sigma }}_{res(ij)}}{\sqrt{{\hat{\sigma }}_{g(i)}^{2}+{\hat{\sigma }}_{res(i)}^{2}}*\sqrt{{\hat{\sigma }}_{g(j)+}^{2}{\hat{\sigma }}_{res(j)}^{2}}},$$

where ${\hat{\sigma }}_{g(ij)}$, is the genetic covariance estimated between progenies for the pair of harvests *i* and *j*; ${\hat{\sigma }}_{g(i)}^{2}$ and ${\hat{\sigma }}_{g(j)}^{2}$ are the genetic variance estimated among progenies of harvests *i* and *j*, respectively; ${\hat{\sigma }}_{res(ij)}$ is the residual covariance estimated between the progenies for the pair of harvests *i* and *j*; and, ${\hat{\sigma }}_{res(i)}^{2}$ and ${\hat{\sigma }}_{res(j)}^{2}$ are the residual variance estimated among progenies of harvests *i* and *j*, respectively.

### Genetic selection based on selection index

The additive index (AI) [22] was used to identify superior families to be selected in the *Jatropha curcas* breeding program. The AI was given by:

$${AI}_{k}=\sum_{h=1}^{6}w_{h}\frac{\left(u_{h}+f_{kh}\right)}{\sigma_{h}},$$

where *w*_*h*_ is the weight assigned for harvest *h*; *u*_*h*_ + *f*_*kh*_ is the overall mean for harvest *h* added to the estimated genetic value of family *k* at harvest *h*; and *σ*_*h*_ is the standard deviation for *u*_*h*_ + *f*_*kh*_. Weights equal to *u*_*h*_/*u*, where *u* is the overall mean, were assigned in the AI.

The selection gains (SG) were obtained directly by the AI output, considering four different selection intensities: 27.4%, 20.5%, 13.7%, and 6.9%, which referred to the selection of 20, 15, 10 and 5 progenies, respectively, as follows:

$$SG(\%)=\left(\frac{X_{s}-X_{o}}{X_{o}}\right)*100.$$

where *X*_*s*_ is the overall mean of the estimated genetic values of the selected progenies, and *X*_*o*_ is the overall mean of the population.

All analyses were carried out in the R software [23], using ‘*MCMCglmm’* [24] and ‘*boa’* [25] packages.

## Results

**Fig 1:**
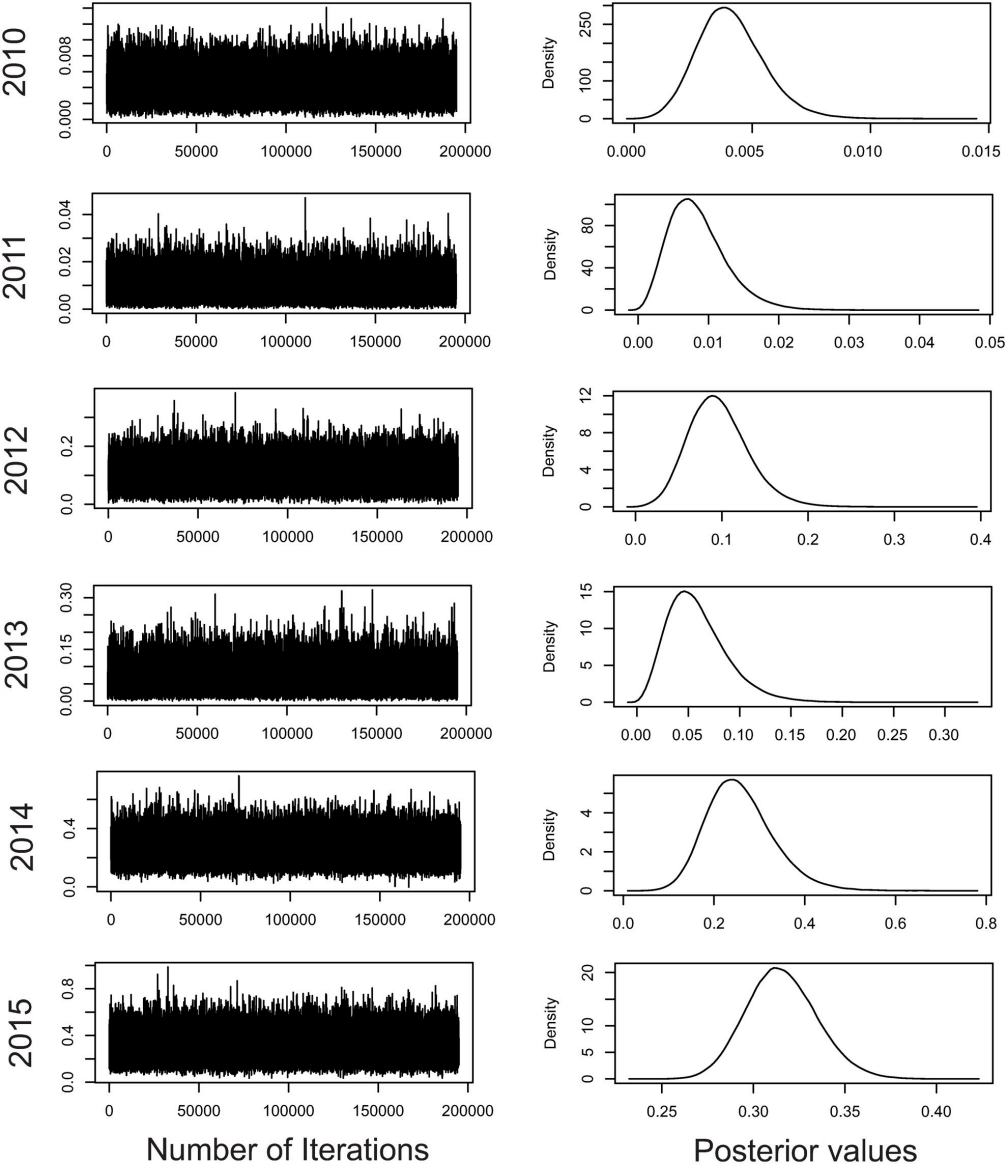
Convergence for the genotypic variance in the six harvests analyzed. The figures on the right refer to the posterior density of the genetic variance estimates. The figures on the left refer to the Markov chains for the genetic variance estimates.

The chains [(co)variance components] achieved convergence by the Geweke criterion, with 180,000 effective samples. Thus, the posterior mean estimates were obtained for the variance components, which suggested density with qui square and normal shape appearances (Fig 1). M1 to M5 present qui square (from which the Wishart distribution is a generalization) and only M6 shows normal appearance. This corroborates the adequacy of the prior generalized qui square used. Only M6 showed statistical learning from the data through the likelihood function. According to the DIC, there was evidence that the full model (DIC = 1399.212) is the one that best fits the data, which reveals the significance of the genotypic effects (DIC = 2742.933) and plot effects (DIC = 1403.41).

The Bayesian MTM led to the estimation of variance components for each environment individually. For the grain yield trait, genetic variances ranged from 0.004 to 0.290 (Table 1). This dissimilarity among harvests was also observed for phenotypic variances (ranging from 0.014 to 0.882) and residual variances (ranging between 0.006 and 0.316). For the 2010, 2012, and 2015 harvests, the coefficients of determination of residual effects was higher than the others. Conversely, the coefficients of determination of plot effects were higher, when compared with the others, in the 2011 and 2013 harvests (0.449 and 0.591, respectively). The heritability estimates were higher ($h_{g}^{2}>0.50$) in the 2010, 2012, 2014, and 2015 harvests, whereas it’s were classified as lower ($h_{g}^{2}<0.29$) in the 2011 and 2013 harvests. Lastly, the HDP intervals indicates significance for all random effects of the MTM (Table 1 and S1 Table).

**Table 1:** Estimate of variance components and genetic and non-genetic parameters for the 73 progenies estimated though MCMC algorithm. The HPD intervals are indicated into the parenthesis.

Harvest	${\hat{\sigma }}_{g}^{2}$	${\hat{\sigma }}_{a}^{2}$	${\hat{\sigma }}_{p}^{2}$	${\hat{\sigma }}_{res}^{2}$	${\hat{\sigma }}_{phen}^{2}$	$h_{g}^{2}$	$c_{p}^{2}$	$c_{res}^{2}$	$r_{\hat{g}g}$	μ
**2010**	0.004	0.016	0.004	0.006	0.014	0.580	0.313	0.400	0.79	0.231
	(0.001–007)	(0.005–0.027)	(0.002–0.006)	(0.005–0.006)	(0.012–0.017)	(0.346–0.781)	(0.179–0.459)	(0.325–0.481)		
**2011**	0.009	0.034	0.033	0.032	0.073	0.292	0.449	0.434	0.88	0.329
	(0.001–0.017)	(0.005–0.067)	(0.022–0.044)	(0.028–0.035)	(0.062–0.086)	(0.087–0.505)	(0.338–0.554)	(0.359–0.511)		
**2012**	0.096	0.385	0.131	0.145	0.372	0.533	0.352	0.390	0.86	1.362
	(0.031–0.166)	(0.121–0.664)	(0.077–0.189)	(0.128–0.162)	(0.309–0.443)	(0.295–0.747)	(0.217–0.496)	(0.318–0.469)		
**2013**	0.059	0.237	0.288	0.139	0.486	0.265	0.591	0.286	0.87	1.240
	(0.01–0.118)	(0.035–0.468)	(0.208–0.376)	(0.123–0.155)	(0.399–0.581)	(0.066–0.460)	(0.481–0.698)	(0.231–0.349)		
**2014**	0.257	1.030	0.207	0.204	0.669	0.663	0.311	0.306	0.85	2.181
	(0.119–0.402)	(0.480–1.611)	(0.124–0.300)	(0.181–0.228)	(0.539–0.815)	(0.487–0.818)	(1.184–0.444)	(1.241–0.377)		
**2015**	0.290	1.159	0.277	0.316	0.882	0.620	0.314	0.361	0.88	2.639
	(0.132–0.467)	(0.514–1.851)	(0.159–0.404)	(0.279–0.354)	(0.720–1.061)	(0.428–0.795)	(1.190–0.449)	(1.286–0.435)		

${\hat{\sigma }}_{g}^{2}$ = genotypic variance; ${\hat{\sigma }}_{a}^{2}$ = additive genetic variance; ${\hat{\sigma }}_{p}^{2}$ = plot variance; ${\hat{\sigma }}_{res}^{2}$ = residual variance; ${\hat{\sigma }}_{phen}^{2}$ = phenotypic variance; $h_{g}^{2}$ = heritability of family means; $c_{p}^{2}$ = coefficient of determination of plot effects; $c_{res}^{2}$ = coefficient of determination of residual effects; $r_{\hat{g}g}$ = selective Bayesian accuracy; and μ: phenotypic mean.

**Fig 2:**
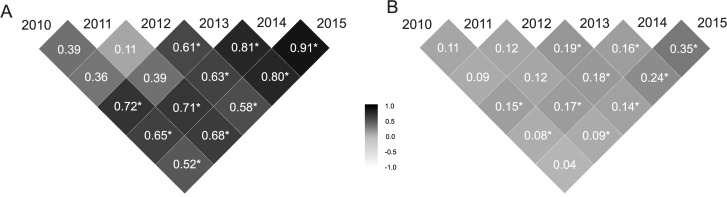
Genetic correlations (A) and phenotypic correlations (B) between pairs of harvests for the 73 progenies of *Jatropha curcas*. The asterisk indicates significance of the correlations coefficients based on the HPD intervals (HPD intervals are presented in S2 Table).

The mean selective accuracies in the six measures presented high magnitudes ($r_{\hat{g}g}>0.70$) (Table 1). Genetic correlations between pairs of harvests (*ρ*_*g*_) presented high values (*ρ*_*g*_ > 0.66) for the 2011–2014, 2010–2013, 2013–2014, 2013–2015 and 2014–2015 pairs of harvests (Fig 2A). On the other hand, the values of genetic correlation between 2011–2012 pair of harvest was lower (*ρ*_*g*_ < 0.33). The remaining values were moderate (0.34 < *ρ*_*g*_ < 0.66). The phenotypic correlation between pairs of harvests (*ρ*_*p*_) was lower (*ρ*_*p*_ < 0.33) in all pairs of harvests, except for the pair 2014–2015, which was moderate (0.34 < *ρ*_*p*_ < 0.66) (Fig 2B).

**Fig 3:**
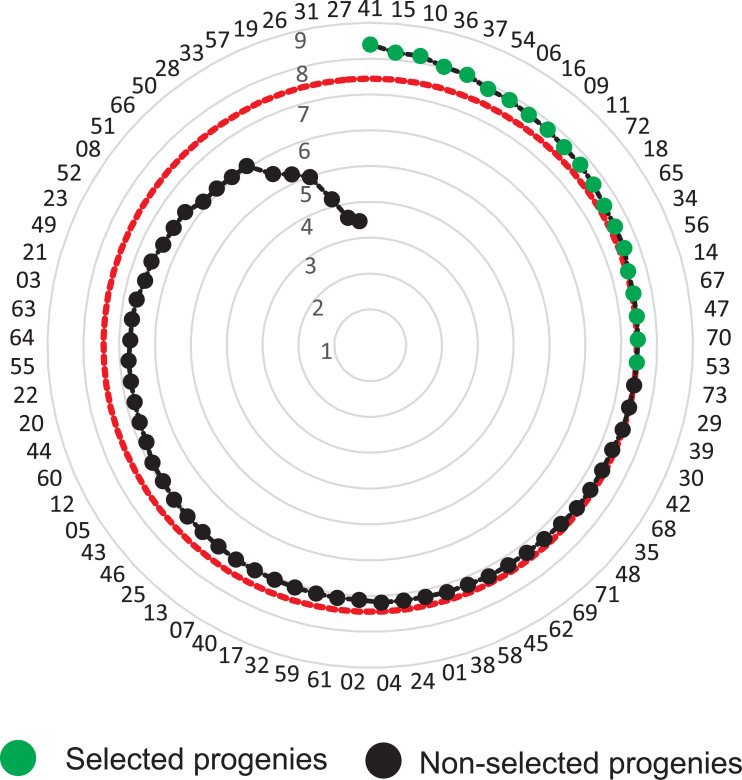
Genetic selection considering the selection intensity of 27% (20 progenies). The red dotted line indicates the selected *Jatropha curcas* progenies.

The additive index indicated a selection gain of 17.29%, when selecting five families, namely, 41, 15, 10, 36 and 37. As expected, the selection gains decreased with the increase of selected families: 10 progenies (SG = 14.74%), 15 progenies (SG = 13.04%), and 20 progenies (SG = 11.60%). The ranking with the 20 progenies selected was presented in Fig 3 and the complete ranking was presented in S3 Table.

## Discussion

The chains convergence ensures that the most likely estimate for each co(variance) component was achieved. The significance of genetic effects indicates genetic variability among the 73 half-sib families, which allows the genetic selection. Deviance information criterion is widely applied as a criterion to evaluate the goodness of fit of models in Bayesian inference [12]. The HPD intervals also indicate the significance of the genetic (progenies) and plot effects.

One advantage of the Bayesian inference in relation with the frequentist inference is the possibility of obtaining the HPD intervals. The HPD intervals are more accurate, when compared with the confidence intervals from frequentist inference, which increases the reliability of the variance components and genetic parameters estimated through Bayesian inference. High values of selective accuracies ($0.70<r_{\hat{g}g}<0.89$) were obtained, indicating high reliability and a favorable scenario for genetic selection since high accuracy allows correct ranking of the genotypes.

It is important to highlight that in this study no prior information was used. Thus, the genetic parameters estimates through Bayesian inference presented similar values those estimated through frequentist inference by restricted maximum likelihood (REML) [26]. In addition, the gain with selection was similar with the MTM frequentist [10], for the same selective intensity (13.49%, referring to 20 progenies).

The HPD intervals and selective accuracies magnitudes demonstrate the reliability and usefulness of the Bayesian inference in *Jatropha curcas* breeding. It is worth mentioning that high selective accuracy directly affects the success of breeding program, since the selective accuracy is the most relevant parameter for evaluation of the effectiveness of the inference about the estimated genetic value of a genotype.

The *Jatropha curcas* progenies evaluated in this study are non-domesticated, with great potential of improvement [1, 5, 27]. However, many studies have pointed out the small genetic variability in this species [7, 28–31]. In this sense, carry out a recurrent selection program is a suitable strategy for the exploration of the additive genetic variability [30, 32]. Another possibility to add genetic variability in the *Jatropha curcas* breeding programs is the addition of new accessions.

The selective accuracies increased in the last two harvests, as well as the genetic and phenotypic correlations, which were higher between the last harvests. As indicated by [33], at least four harvests (years) are needed for the genetic selection of superior *Jatropha curcas* genotypes for the grain yield trait.

The heritability estimates present a fluctuating pattern, going up and down from one measure to another. Such trend could be related with the genotype × year interactions, especially in the early stages of growth, where the plants are more sensitive to environmental variations, and to the fact that the metabolism of young perennials often benefit the vegetative over reproductive growth, implying in an uneven genetic response in the early stages [5, 34]. These results indicate a biannual variation of the *Jatropha curcas* genotypes.

Two aspects in which the Bayesian MTM can outperform the frequentist MTM should be highlighted: (i) it provides the HPD intervals [35], and (ii) flexibility in choosing the distributions for the data set and unknown parameters and the possibility of incorporating prior knowledge about the parameters of the model [36, 37]. It is worth mentioning that the REML method also presents hindrances regarding the convergence of the MTM [38], whereas the convergence of the Bayesian MTM it is easier to achieve [11]. Therefore, when compared with the frequentist inference, it seems reasonable that the Bayesian inference presents better results [11, 13, 14, 39, 40].

## Conclusion

The Bayesian MTM revealed to be a suitable strategy for repeated measures analysis in *Jatropha curcas* breeding. Besides that, the MTM have potential for genetic evaluation of any perennial.

## Supporting information

S1 Table High posterior density (HPD) intervals of the variance and covariance components for the G, T, and R matrices of the multiple-trait Bayesian model. VCV: variances and covariances between pairs of harvests. M1 to M6 represents the six harvests (2010 to 2015).
(DOCX)

S2 Table High posterior density (HPD) intervals for the genetic and phenotypic correlations between pairs of harvests (M1 to M6). The asterisk represents those estimates that are significant by HPD intervals.
(DOCX)

S3 Table Additive index (AI), selection gain (SG), and selection gain in percentage (SG%), based on the estimated genetic values of the 73 *Jatropha curcas* progenies. (DOCX)
