# Supplementary material for: Multiple-trait model through Bayesian inference applied to Jatropha curcas breeding for bioenergy
Source: PLoS One. 2021 Mar 4;16(3):e0247775. doi: 10.1371/journal.pone.0247775 (PMC7932130; doi:10.1371/journal.pone.0247775)
Supplement: S1 Table — VCV: variances and covariances between pairs of harvests. M1 to M6 represents the six harvests (2010 to 2015). (DOCX) [file pone.0247775.s001.docx]

**S1 Table**. High posterior density (HPD) intervals of the variance and covariance components for the **G**, **T**, and **R** matrices of the multiple-trait Bayesian model. VCV: variances and covariances between pairs of harvests. M1 to M6 represents the six harvests (2010 to 2015).

|  | **Genetic effect** | | **Plot effect** | | **Residual effect** | |
| --- | --- | --- | --- | --- | --- | --- |
| **VCV** | **Lower** | **Upper** | **Lower** | **Upper** | **Lower** | **Upper** |
| **M1:M1** | 0.0015 | 0.0069 | 0.0025 | 0.0067 | 0.0050 | 0.0064 |
| **M2:M1** | -0.0013 | 0.0059 | -0.0027 | 0.0042 | 0.0024 | 0.0047 |
| **M3:M1** | -0.0028 | 0.0176 | -0.0004 | 0.0161 | 0.0048 | 0.0097 |
| **M4:M1** | 0.0014 | 0.0216 | -0.0043 | 0.0151 | 0.0036 | 0.0084 |
| **M5:M1** | 0.0063 | 0.0366 | -0.0044 | 0.0157 | 0.0014 | 0.0071 |
| **M6:M1** | 0.0019 | 0.0344 | -0.0101 | 0.0135 | -0.0006 | 0.0066 |
| **M1:M2** | -0.0013 | 0.0059 | -0.0027 | 0.0042 | 0.0024 | 0.0047 |
| **M2:M2** | 0.0014 | 0.0166 | 0.0228 | 0.0445 | 0.0283 | 0.0357 |
| **M3:M2** | -0.0133 | 0.0214 | 0.0069 | 0.0440 | 0.0213 | 0.0334 |
| **M4:M2** | -0.0078 | 0.0243 | 0.0003 | 0.0444 | 0.0146 | 0.0262 |
| **M5:M2** | 0.0054 | 0.0613 | 0.0074 | 0.0552 | 0.0129 | 0.0268 |
| **M6:M2** | 0.0056 | 0.0634 | -0.0183 | 0.0344 | 0.0050 | 0.0219 |
| **M1:M3** | -0.0028 | 0.0176 | -0.0004 | 0.0161 | 0.0048 | 0.0097 |
| **M2:M3** | -0.0133 | 0.0214 | 0.0069 | 0.0440 | 0.0213 | 0.0334 |
| **M3:M3** | 0.0311 | 0.1658 | 0.0770 | 0.1892 | 0.1284 | 0.1621 |
| **M4:M3** | -0.0016 | 0.0965 | -0.0160 | 0.0836 | 0.0305 | 0.0551 |
| **M5:M3** | 0.0239 | 0.1805 | 0.0159 | 0.1253 | 0.0356 | 0.0651 |
| **M6:M3** | 0.0183 | 0.1822 | -0.0229 | 0.0990 | 0.0333 | 0.0703 |
| **M1:M4** | 0.0014 | 0.0216 | -0.0043 | 0.0151 | 0.0036 | 0.0084 |
| **M2:M4** | -0.0078 | 0.0243 | 0.0003 | 0.0444 | 0.0146 | 0.0262 |
| **M3:M4** | -0.0016 | 0.0965 | -0.0160 | 0.0836 | 0.0305 | 0.0551 |
| **M4:M4** | 0.0095 | 0.1183 | 0.2085 | 0.3762 | 0.1233 | 0.1556 |
| **M5:M4** | 0.0256 | 0.1780 | 0.0019 | 0.1309 | 0.0193 | 0.0476 |
| **M6:M4** | 0.0226 | 0.1959 | 0.0947 | 0.2586 | 0.0437 | 0.0810 |
| **M1:M5** | 0.0063 | 0.0366 | -0.0044 | 0.0157 | 0.0014 | 0.0071 |
| **M2:M5** | 0.0054 | 0.0613 | 0.0074 | 0.0552 | 0.0129 | 0.0268 |
| **M3:M5** | 0.0239 | 0.1805 | 0.0159 | 0.1253 | 0.0356 | 0.0651 |
| **M4:M5** | 0.0256 | 0.1780 | 0.0019 | 0.1309 | 0.0193 | 0.0476 |
| **M5:M5** | 0.1187 | 0.4016 | 0.1241 | 0.3004 | 0.1812 | 0.2288 |
| **M6:M5** | 0.1228 | 0.3826 | -0.0145 | 0.1376 | 0.0752 | 0.1214 |
| **M1:M6** | 0.0019 | 0.0344 | -0.0101 | 0.0135 | -0.0006 | 0.0066 |
| **M2:M6** | 0.0056 | 0.0634 | -0.0183 | 0.0344 | 0.0050 | 0.0219 |
| **M3:M6** | 0.0183 | 0.1822 | -0.0229 | 0.0990 | 0.0333 | 0.0703 |
| **M4:M6** | 0.0226 | 0.1959 | 0.0947 | 0.2586 | 0.0437 | 0.0810 |
| **M5:M6** | 0.1228 | 0.3826 | -0.0145 | 0.1376 | 0.0752 | 0.1214 |
| **M6:M6** | 0.1315 | 0.4670 | 0.1595 | 0.4047 | 0.2791 | 0.3543 |
