# Supplementary material for: Multiple-trait model through Bayesian inference applied to Jatropha curcas breeding for bioenergy
Source: PLoS One. 2021 Mar 4;16(3):e0247775. doi: 10.1371/journal.pone.0247775 (PMC7932130; doi:10.1371/journal.pone.0247775)
Supplement: S2 Table — The asterisk represents those estimates that are significant by HPD intervals. (DOCX) [file pone.0247775.s002.docx]

**S2 Table**. High posterior density (HPD) intervals for the genetic and phenotypic correlations between pairs of harvests (M1 to M6). The asterisk represents those estimates that are significant by HPD intervals.

| Pair | Genetic | | Phenotypic | |
| --- | --- | --- | --- | --- |
|  | Inferior | Superior | Inferior | Superior |
| **M1:M2** | -0.1313 | 0.9849 | -0.0517 | 0.2636 |
| **M1:M3** | -0.1262 | 0.8024 | -0.0349 | 0.2121 |
| **M1:M4** | 0.3113* | 0.9995* | 0.0475* | 0.2583* |
| **M1:M5** | 0.3401* | 0.9821* | 0.0153* | 0.1493* |
| **M1:M6** | 0.1305* | 0.9561* | -0.0104 | 0.0984 |
| **M2:M3** | -0.5061 | 0.7285 | -0.4366 | 0.6299 |
| **M2:M4** | -0.2176 | 0.9931 | -0.0836 | 0.3141 |
| **M2:M5** | 0.3408* | 0.9983* | 0.0651* | 0.2806* |
| **M2:M6** | 0.2762* | 0.9985* | 0.0124* | 0.1720* |
| **M3:M4** | 0.1384* | 0.9988* | 0.0249* | 0.3305* |
| **M3:M5** | 0.3185* | 0.9217* | 0.0803* | 0.2870* |
| **M3:M6** | 0.2270* | 0.9402* | 0.0416* | 0.2445* |
| **M4:M5** | 0.4933* | 0.9997* | 0.0708* | 0.2505* |
| **M4:M6** | 0.5092* | 0.9997* | 0.1257* | 0.3444* |
| **M5:M6** | 0.7612* | 0.9999* | 0.2619* | 0.4395* |
