# Supplementary material for: Multiple-trait model through Bayesian inference applied to Jatropha curcas breeding for bioenergy
Source: PLoS One. 2021 Mar 4;16(3):e0247775. doi: 10.1371/journal.pone.0247775 (PMC7932130; doi:10.1371/journal.pone.0247775)
Supplement: S3 Table — (DOCX) [file pone.0247775.s003.docx]

**S3 Table.** Additive index (AI), selection gain (SG), and selection gain in percentage (SG%), based on the estimated genetic values of the 73 *Jatropha curcas* progenies.

| **Progeny** | **2010** | **2011** | **2012** | **2013** | **2014** | **2015** | **AI** | **SG** | **SG(%)** |
| --- | --- | --- | --- | --- | --- | --- | --- | --- | --- |
| **41** | 0.326 | 0.644 | 1.775 | 1.765 | 3.078 | 3.489 | 8.394 | 1.206 | 20.555 |
| **15** | 0.311 | 0.566 | 2.060 | 1.731 | 2.912 | 3.225 | 8.199 | 1.192 | 19.154 |
| **10** | 0.244 | 0.546 | 2.135 | 1.678 | 2.907 | 3.281 | 8.191 | 1.186 | 18.649 |
| **36** | 0.313 | 0.651 | 1.676 | 1.668 | 2.936 | 3.352 | 8.032 | 1.178 | 17.827 |
| **37** | 0.273 | 0.657 | 1.659 | 1.635 | 2.946 | 3.408 | 8.020 | 1.173 | 17.297 |
| **54** | 0.262 | 0.592 | 1.823 | 1.637 | 2.776 | 3.274 | 7.873 | 1.166 | 16.592 |
| **6** | 0.262 | 0.590 | 1.782 | 1.666 | 2.789 | 3.260 | 7.865 | 1.161 | 16.072 |
| **16** | 0.282 | 0.535 | 1.938 | 1.695 | 2.740 | 3.073 | 7.801 | 1.156 | 15.569 |
| **9** | 0.220 | 0.613 | 1.804 | 1.547 | 2.837 | 3.243 | 7.789 | 1.152 | 15.156 |
| **11** | 0.251 | 0.627 | 1.690 | 1.570 | 2.759 | 3.282 | 7.730 | 1.147 | 14.742 |
| **72** | 0.321 | 0.617 | 1.626 | 1.662 | 2.783 | 3.171 | 7.727 | 1.144 | 14.400 |
| **18** | 0.273 | 0.560 | 1.824 | 1.624 | 2.736 | 3.086 | 7.672 | 1.140 | 14.049 |
| **65** | 0.245 | 0.579 | 1.824 | 1.558 | 2.606 | 3.180 | 7.599 | 1.137 | 13.671 |
| **34** | 0.246 | 0.641 | 1.643 | 1.533 | 2.709 | 3.218 | 7.589 | 1.133 | 13.337 |
| **56** | 0.246 | 0.546 | 1.793 | 1.628 | 2.670 | 3.093 | 7.586 | 1.130 | 13.044 |
| **14** | 0.261 | 0.616 | 1.673 | 1.525 | 2.700 | 3.083 | 7.484 | 1.127 | 12.697 |
| **67** | 0.252 | 0.513 | 1.881 | 1.622 | 2.516 | 3.034 | 7.477 | 1.124 | 12.385 |
| **47** | 0.227 | 0.579 | 1.776 | 1.524 | 2.633 | 3.093 | 7.473 | 1.121 | 12.103 |
| **70** | 0.272 | 0.708 | 1.208 | 1.574 | 2.768 | 3.300 | 7.465 | 1.118 | 11.846 |
| **53** | 0.226 | 0.558 | 1.755 | 1.558 | 2.538 | 3.147 | 7.446 | 1.116 | 11.601 |
| **73** | 0.239 | 0.589 | 1.606 | 1.579 | 2.627 | 3.139 | 7.439 | 1.114 | 11.374 |
| **29** | 0.197 | 0.556 | 1.853 | 1.508 | 2.555 | 3.090 | 7.427 | 1.112 | 11.159 |
| **39** | 0.250 | 0.601 | 1.565 | 1.586 | 2.642 | 3.111 | 7.419 | 1.110 | 10.959 |
| **30** | 0.330 | 0.590 | 1.554 | 1.624 | 2.658 | 2.930 | 7.355 | 1.107 | 10.737 |
| **42** | 0.228 | 0.521 | 1.873 | 1.554 | 2.503 | 2.978 | 7.352 | 1.105 | 10.531 |
| **68** | 0.266 | 0.585 | 1.595 | 1.580 | 2.547 | 3.082 | 7.348 | 1.103 | 10.339 |
| **35** | 0.230 | 0.613 | 1.604 | 1.506 | 2.593 | 3.105 | 7.339 | 1.102 | 10.156 |
| **48** | 0.213 | 0.625 | 1.500 | 1.506 | 2.609 | 3.131 | 7.291 | 1.100 | 9.961 |
| **71** | 0.304 | 0.616 | 1.458 | 1.583 | 2.576 | 3.014 | 7.262 | 1.098 | 9.766 |
| **69** | 0.243 | 0.560 | 1.681 | 1.532 | 2.522 | 3.003 | 7.257 | 1.096 | 9.582 |
| **62** | 0.209 | 0.568 | 1.742 | 1.480 | 2.453 | 3.061 | 7.245 | 1.094 | 9.403 |
| **45** | 0.221 | 0.536 | 1.745 | 1.534 | 2.485 | 2.993 | 7.244 | 1.092 | 9.236 |
| **58** | 0.260 | 0.583 | 1.541 | 1.549 | 2.534 | 3.020 | 7.217 | 1.091 | 9.066 |
| **38** | 0.233 | 0.581 | 1.608 | 1.508 | 2.514 | 3.038 | 7.214 | 1.089 | 8.906 |
| **1** | 0.228 | 0.592 | 1.553 | 1.525 | 2.544 | 3.007 | 7.192 | 1.087 | 8.745 |
| **24** | 0.331 | 0.564 | 1.549 | 1.645 | 2.532 | 2.821 | 7.185 | 1.086 | 8.591 |
| **4** | 0.194 | 0.675 | 1.471 | 1.382 | 2.601 | 3.130 | 7.184 | 1.084 | 8.445 |
| **2** | 0.211 | 0.691 | 1.445 | 1.356 | 2.592 | 3.075 | 7.117 | 1.083 | 8.281 |
| **61** | 0.205 | 0.547 | 1.726 | 1.482 | 2.376 | 2.995 | 7.113 | 1.081 | 8.123 |
| **59** | 0.209 | 0.631 | 1.394 | 1.428 | 2.502 | 3.164 | 7.093 | 1.080 | 7.967 |
| **32** | 0.290 | 0.649 | 1.287 | 1.532 | 2.561 | 2.988 | 7.077 | 1.078 | 7.813 |
| **17** | 0.249 | 0.609 | 1.524 | 1.466 | 2.520 | 2.922 | 7.064 | 1.077 | 7.661 |
| **40** | 0.247 | 0.561 | 1.675 | 1.494 | 2.443 | 2.857 | 7.061 | 1.075 | 7.516 |
| **7** | 0.234 | 0.575 | 1.605 | 1.467 | 2.505 | 2.887 | 7.052 | 1.074 | 7.374 |
| **13** | 0.226 | 0.541 | 1.720 | 1.473 | 2.435 | 2.841 | 7.031 | 1.072 | 7.232 |
| **25** | 0.291 | 0.574 | 1.428 | 1.571 | 2.484 | 2.853 | 7.002 | 1.071 | 7.087 |
| **46** | 0.218 | 0.543 | 1.621 | 1.486 | 2.396 | 2.906 | 6.986 | 1.069 | 6.943 |
| **43** | 0.243 | 0.506 | 1.724 | 1.525 | 2.376 | 2.775 | 6.971 | 1.068 | 6.801 |
| **5** | 0.168 | 0.504 | 1.933 | 1.389 | 2.293 | 2.780 | 6.914 | 1.066 | 6.647 |
| **12** | 0.255 | 0.511 | 1.757 | 1.488 | 2.339 | 2.704 | 6.897 | 1.065 | 6.495 |
| **60** | 0.220 | 0.504 | 1.665 | 1.482 | 2.256 | 2.788 | 6.803 | 1.063 | 6.323 |
| **44** | 0.240 | 0.547 | 1.573 | 1.455 | 2.331 | 2.756 | 6.782 | 1.062 | 6.151 |
| **20** | 0.180 | 0.526 | 1.678 | 1.391 | 2.329 | 2.772 | 6.762 | 1.060 | 5.981 |
| **22** | 0.229 | 0.581 | 1.497 | 1.412 | 2.369 | 2.780 | 6.753 | 1.058 | 5.814 |
| **55** | 0.280 | 0.533 | 1.551 | 1.497 | 2.293 | 2.707 | 6.752 | 1.057 | 5.654 |
| **64** | 0.213 | 0.505 | 1.672 | 1.450 | 2.165 | 2.760 | 6.695 | 1.055 | 5.484 |
| **63** | 0.180 | 0.562 | 1.525 | 1.365 | 2.239 | 2.896 | 6.687 | 1.053 | 5.318 |
| **3** | 0.206 | 0.602 | 1.483 | 1.338 | 2.331 | 2.760 | 6.640 | 1.051 | 5.146 |
| **21** | 0.194 | 0.495 | 1.646 | 1.407 | 2.213 | 2.613 | 6.537 | 1.050 | 4.955 |
| **49** | 0.250 | 0.525 | 1.569 | 1.428 | 2.197 | 2.592 | 6.530 | 1.048 | 4.769 |
| **23** | 0.185 | 0.556 | 1.389 | 1.331 | 2.202 | 2.752 | 6.415 | 1.046 | 4.562 |
| **52** | 0.179 | 0.533 | 1.507 | 1.342 | 2.133 | 2.664 | 6.383 | 1.044 | 4.354 |
| **8** | 0.189 | 0.606 | 1.283 | 1.285 | 2.269 | 2.725 | 6.363 | 1.041 | 4.148 |
| **51** | 0.172 | 0.458 | 1.661 | 1.330 | 1.982 | 2.427 | 6.143 | 1.039 | 3.899 |
| **66** | 0.194 | 0.529 | 1.336 | 1.325 | 2.047 | 2.585 | 6.123 | 1.037 | 3.653 |
| **50** | 0.198 | 0.523 | 1.392 | 1.325 | 2.016 | 2.493 | 6.076 | 1.034 | 3.405 |
| **28** | 0.208 | 0.526 | 1.404 | 1.325 | 2.028 | 2.455 | 6.073 | 1.032 | 3.163 |
| **33** | 0.171 | 0.467 | 1.376 | 1.206 | 1.813 | 2.143 | 5.494 | 1.028 | 2.807 |
| **57** | 0.149 | 0.507 | 1.171 | 1.118 | 1.651 | 2.239 | 5.243 | 1.024 | 2.408 |
| **19** | 0.198 | 0.451 | 1.210 | 1.154 | 1.546 | 1.929 | 4.984 | 1.020 | 1.968 |
| **26** | 0.131 | 0.376 | 1.224 | 1.003 | 1.196 | 1.512 | 4.211 | 1.014 | 1.383 |
| **31** | 0.112 | 0.371 | 1.010 | 0.900 | 0.944 | 1.299 | 3.612 | 1.007 | 0.696 |
| **27** | 0.122 | 0.397 | 0.866 | 0.856 | 0.943 | 1.278 | 3.475 | 1.000 | 0.000 |
